# Editorial: COVID-19: Mid- and Long-Term Educational and Psychological Consequences for Students and Educators

**DOI:** 10.3389/fpsyg.2022.903022

**Published:** 2022-04-18

**Authors:** Maria Jose Alvarez-Alonso, Ricardo Scott, Isabel Morales-Muñoz

**Affiliations:** ^1^Departamento de Educación, Universidad Nebrija, Madrid, Spain; ^2^Departamento de Psicología Evolutiva y Didáctica, Universidad de Alicante, San Vicente del Raspeig, Spain; ^3^Institute for Mental Health, School of Psychology, University of Birmingham, Edgbaston, United Kingdom

**Keywords:** COVID-19, education, mental health-related quality of life, psychological consequences, mid and long term consequences

## Introduction

COVID-19 pandemic has been a great challenge to our societies. To reduce its impact, different approaches, and politics have been used. However, public health, along with educational breaches have been evidenced in most countries in which not all citizens have the same opportunities to deal with the pandemic. Therefore, this has led to pervasive consequences, including mental health problems because of the disruption of everyday life routines.

## Literature and Research

The impact of COVID-19 pandemic on the educational system happened abruptly in the spring of 2020, when most of the countries experienced a shift from face-to-face education to distance and online learning, or in the worst cases, to non-existent education. The consequences of such changes have been studied at the different stages of the education within this Research Topic (Figueroa-Quiñones et al., in university students; Rao and Rao, in highschoolers; Combette et al. in middle school scholars; or even honors research students in Kumar et al.).

In countries where the population did not have the possibility to get access to electronic devices or the internet, governments implemented alternative educational strategies, including teaching via public TV channels or even cell phone applications, such as *WhatsApp or Telegram* to set homework for children. In other cases, school platforms were used so that teachers and students were able to interact, but this was only possible in those schools where digitalization and teacher training was already initiated (do Amaral et al., 2021). These changes once again demonstrated the lack of equity in access to education, not only between countries, but also within-countries. In February 2022, some regions in the world still have partially opened schools and educational centers, affecting more than 40 million enrolled learners around the world (United Nations Educational Scientific and Cultural Organization, 2022).

The existing resources are not the same for everyone, and even the closure of schools showed the bitter face of the reality of many children, who were not only left without an education, but without their daily food rations (World Food Program, 2020). Further, some other children were forced to stay at home with their aggressors and abusers, as described in the opinion article by Fares-Otero and Trautmann, published in this Research Topic.

Hardly any country knew how to act ahead of what was happening in different parts of the world, leaving populations at their mercy until the healthcare system collapsed. Governments began then to impose lockdowns, restrictions on mobility, curfews, and prohibitions, along with the closure of workplaces and non-essential activities, including schools. Children and adolescents were confined to their homes with no other alternatives. Even currently (March 2022), in some countries there are still restrictions on children's access to education because of the pandemic. For instance, most children and adolescents, and their teachers, must wear masks for several hours in their classes, something which is not only uncomfortable, but also impedes a natural social interaction because they cannot see each other's facial expressions. In addition, many social activities are still restricted and consequently, all these changes in everyday lives and activities have a clear impact on mental health.

Many of the problems that we have been experiencing during the last 2 years of the pandemic have been described in this Research Topic as a result of the authors' examination of the ongoing reality in their countries. Undoubtedly, their research helps us to better understand the repercussions that the pandemic will have, not only in terms of mental health consequences, but also in terms of education, equal opportunities and/or access to health, education, work, and quality social networks, which are all essential factors for the integral development of the human being.

Two years later, we have seen that the pandemic has brought stress, anxiety, depression, and symptomatology associated with post-traumatic stress in a broad spectrum of the population. The frequency of emotional disorders has considerably increased in children, adolescents, and young people, who have seen their development and their freedom impeded. In addition, eating disorders in children and younger adolescents have multiplied, and self-injurious behaviors and suicidal ideations have also notoriously increased and are extremely common, reaching, in some countries alarming figures (Henry et al., 2021). Despite this dramatic situation, the policies of most countries in relation to mental health have not changed much.

In this Topic Research, Rao and Rao studied mental health and stress in teens at High School, reporting a positive correlation between mental health degradation and online learning, with a negative correlation between physical exercise and mental health degradation. These findings are important because in many countries, for more than one scholar-year, all classes were conducted online, and thus physical exercise was not possible due to the pandemic restrictions.

Another relevant factor is that the resources for education during the pandemic have not changed significantly in many countries either. Therefore, the attention to students remained similar, although with stressed and burned-out professionals. In different parts of the world, and in different cultures, we have observed very similar reactions from the population, witnessing a global decline in mental health in young people, which will undoubtedly have repercussions in the future. The research works included in this Topic Research address different examples of this kind.

Educational resources are low in many countries and regions. Not all the students or teachers have access to an adequate e-learning environment, or access to computers and/or internet and, above all, training in digital competencies are still not guaranteed in most places. Weißenfels et al. investigated the burnout and self-efficacy in teachers during the first pandemic school year in Germany, a country where the access to internet is common [e.g., 94% of internet use, according to the Federal Statistical Office of Germany (2022). https://www.destatis.de] and the access to the internet is considered a basic right. However, even under these favorable circumstances, in Germany, the lack of educational resources was still considered a great challenge to the education professionals, who experienced problems with their daily activities.

Finally, it is important to define protective factors which could ameliorate the negative impact that the pandemic has exerted in our daily lives. For instance, in the study conducted by Combette et al. the authors identified the student's motivation as a key factor for engagement throughout difficult times because of the pandemic, in middle school students. Further, some ideas to prevent dropout or failure in the event of a catastrophe (i.e., global or local), have been pointed out by Supriya et al. Among these, we would like to highlight the importance to build a fluid and robust channel of communication between the agents of the educational community so that, in case of need for change or flexibility of the methods, these can have a positive outcome and be adapted to the needs of all.

## Conclusions

In conclusion, this Research Topic highlights the importance of Education and Mental Health after the breakage of the COVID-19 pandemic worldwide. Communication, training, resources, and motivation are all key factors in dealing with such challenging times, but behavioral-educational factors should be also considered, as these are essential if preventive and rationale intervention strategies want to be implemented among the general population (De la Fuente et al., 2021).

Future lines of research should investigate how specific policies and strategies employed in different countries might have a differential impact on the mental health and the educational systems. Further, the long-term effects associated with the COVID-19 pandemic need further consideration. We believe that a detailed examination of the short, mid, and long-term consequences caused by COVID-19 would help us to better prepare for future challenges and to ameliorate their impact.

## Author Contributions

MA-A, RS, and IM-M contributed to the conception, design, writing, and correction of this editorial. All authors contributed to the article and approved the submitted version.

## Conflict of Interest

The authors declare that the research was conducted in the absence of any commercial or financial relationships that could be construed as a potential conflict of interest.

## Publisher's Note

All claims expressed in this article are solely those of the authors and do not necessarily represent those of their affiliated organizations, or those of the publisher, the editors and the reviewers. Any product that may be evaluated in this article, or claim that may be made by its manufacturer, is not guaranteed or endorsed by the publisher.
